# Outpatient CAR T-Cell Therapy as Standard of Care: Current Perspectives and Considerations

**DOI:** 10.46989/001c.115793

**Published:** 2024-04-09

**Authors:** Katie Gatwood, Zahra Mahmoudjafari, Brittney Baer, Stacy Pak, Brian Lee, Hoim Kim, Karin Abernathy, Bhagirathbhai Dholaria, Olalekan Oluwole

**Affiliations:** 1 Pharmacy Vanderbilt University Medical Center; 2 Pharmacy The University of Kansas Cancer Center; 3 Vanderbilt University Medical Center; 4 Pharmacy City Of Hope National Medical Center; 5 City of Hope; 6 City Of Hope National Medical Center; 7 Pharmacy Sarah Cannon Research Institute; 8 Medicine Hematology Oncology Vanderbilt University Medical Center; 9 MedicineHematology and oncology Vanderbilt University Medical Center

**Keywords:** cellular therapy, ambulatory care, CAR T-cell, outpatient

## Abstract

Chimeric antigen receptor T-cell therapy (CAR-T) has altered the treatment landscape of several hematologic malignancies. Until recently, most CAR-T infusions have been administered in the inpatient setting, due to their toxicity profile. However, the advent of new product constructs, as well as improved detection and management of adverse effects, have greatly increased the safety in administering these therapies. CAR-T indications continue to expand, and inpatient administration is associated with increased healthcare resource utilization and overall cost. Therefore, transitioning CAR-T administration to the outpatient setting has been of great interest in an effort to improve access, reduce financial burden, and improve patient satisfaction. Establishment of a successful outpatient CAR-T requires several components, including a multidisciplinary cellular therapy team and an outpatient center with appropriate clinical space and personnel. Additionally, clear criteria for outpatient administration eligibility and for inpatient admission with pathways for prompt toxicity evaluation and admission, and toxicity management guidelines should be implemented. Education about CAR-T therapy and its associated toxicities is imperative for all clinical staff, as well as patients and their caregivers. Finally, rigorous financial planning and close collaboration with payers to ensure equitable access, while effectively managing cost, are essential to program success and sustainability. This review provides a summary of currently published experiences, as well as expert opinion regarding implementation of an outpatient CAR-T program.

## Introduction

Chimeric antigen receptor T-cell therapy (CAR-T) has shown substantial efficacy in the treatment of several types of hematologic malignancies.^1–12^ There are now multiple FDA-approved CAR-T products available for commercial use, which has resulted in a persistent and rapid evolution of the treatment paradigm for high-grade non-Hodgkin lymphoma, acute lymphoblastic leukemia, and multiple myeloma.^13–18^ Given the growth in the clinical application of these therapies, interest in outpatient administration has also intensified as a mechanism to improve CAR-T accessibility and reduce overall health care resource utilization. Although most initial clinical trials supporting CAR-T use were conducted in the inpatient setting, there is now a growing body of real-world evidence supporting the administration of these products in the ambulatory setting. These studies have demonstrated that CAR-T therapy toxicity is similar to or, in some cases, less frequent or severe than that observed in the registration trials. Although the majority of patients in those studies required an inpatient admission for toxicity management, there remains a subset who can remained in the outpatient setting for the entirety of their treatment course.^19–26^ Even among those outpatients who required admission, the median hospital length of stay was approximately 10 days shorter, as compared to patients who received inpatient cell infusion.^22^

With the increasing application and usage of CAR-T products comes more familiarity and, as such, substantial improvements in managing their unique toxicities have been made over the past several years.^27–29^ Additionally, CAR-T manufacturers are making efforts to engineer products with improved safety profiles.^1^ Therefore, it is anticipated that administration of CAR-T therapy will eventually be a standard outpatient procedure. Many centers throughout the USA are currently in the process of establishing outpatient cellular therapy programs to meet this need. There are multiple challenges and factors to consider (Table 1). A successful outpatient program will understand all potential complications of each CAR-T product and be adept at predicting their onset as well as have detailed procedures for prompt recognition and intervention. It will also have the capacity for daily patient monitoring and treatment, a core multidisciplinary team specializing in CAR-T therapy, resources for comprehensive patient education and caregiver support, and the ability to identify or support lodging, so patients can be within the necessary proximity to the treatment center.^19–21,30–33^ This review serves to provide a summary of currently published experiences, as well as expert opinion regarding implementation of an outpatient CAR-T therapy program. These principles are generally applicable across all current commercially approved products and associated disease states.

**Table 1.** Considerations for Implementation of an Outpatient CAR-T Program^31–33^

**Institutional Considerations**	
Regulatory requirements	Hospital and associated clinics certified and enrolled in a REMS program REMS certification of staff Tocilizumab availability Adverse effect reporting structure
Infrastructure/transitions of care	Outpatient clinic operation 7 days/week Telemedicine implementation 24/7 on-call CAR T-cell team/provider Algorithms and/or SOPs for toxicity management, including indication for admission and admission workflows Reserved bed availability Emergency department team education Electronic medical record treatment plans and flowsheets
Patient communication and support	Patient and caregiver education Wearable vital sign monitoring devices Telemedicine implementation

Abbreviations: REMS: Risk Evaluation and Mitigation Strategy; SOP: standard operating procedure; CAR: Chimeric Antigen Receptor

## Outpatient CAR-T Therapy Program Implementation

The success of implementing an outpatient CAR-T therapy program necessitates meticulous financial planning and close collaboration with payers, to ensure equitable access, while effectively managing costs. The critical components and considerations involved in establishing and sustaining outpatient CAR-T therapy programs include awareness and planning for FDA and Risk Evaluation and Mitigation Strategies (REMS) requirements, financial considerations, personnel requirements, infrastructure needs, and metrics for program success.

### FDA and REMS Considerations

The FDA has approved several CAR-T therapies, each with specific REMS programs to ensure safe administration and management of adverse events.^13–18^ While the REMS requirements do not specifically address the site of CAR-T administration, authorized treatment centers must demonstrate an understanding of the requirements, including proximity to the treatment center, patient education, and diligent adverse event monitoring.

### Financial Considerations

Outpatient CAR-T therapy programs require diligent financial planning and coordination due to the intricate and costly nature of CAR-T treatments. Establishing a dedicated reimbursement team or enlisting the services of a reimbursement specialist is imperative for navigating the intricacies of CAR-T reimbursement mechanisms and optimizing financial sustainability. This planning encompasses several critical considerations, including reimbursement mechanisms, patient assistance programs, and the allocation of resources in a cost-effective manner. It is imperative to develop a comprehensive budget that accounts for all expenses associated with the administration of outpatient CAR-T therapy. These expenses span from drug acquisition costs and healthcare provider fees to facility charges and ancillary services. Conducting a detailed cost-benefit analysis to evaluate the financial feasibility of transitioning CAR-T therapies to an outpatient setting is equally crucial, and should involve a thorough comparison with inpatient care to ensure long-term financial sustainability. Revenue sources, exploration of potential cost-saving opportunities, and strategies to effectively manage the financial risks associated with outpatient CAR-T therapy programs are vital to programmatic success. Continual monitoring and adaptation of financial strategies remain essential to support the long-term viability of outpatient CAR-T therapy programs.

Key team members should actively engage with payers to negotiate favorable reimbursement terms, while gaining a clear understanding of reimbursement rates, coding procedures, and billing requirements. The American Society for Transplantation and Cellular Therapy (ASTCT) provides a comprehensive billing and coding guide to aid institutions in coding and billing.^34^ Notably, Medicare and certain private insurers employ Diagnosis-Related Group (DRG) or Ambulatory Payment Classification (APC) systems for determining reimbursement rates in inpatient and outpatient care settings, respectively. Hospital inpatient reimbursement is calculated using the MS-DRG base payment rate adjusted for hospital geography, diagnosis, case severity and discharge status.^35^ APC codes vary based on procedure complexity and cost, making it vital to understand how CAR-T therapy is categorized within these systems. With outpatient administration, Medicare typical reimbursement is based on the hospital outpatient prospective payment system (OPPS). Rigorous adherence to accurate coding and billing practices is essential to maximize reimbursement under the OPPS. Medicaid reimbursement rates are state-dependent and may also be contingent on the specific CAR-T product, patient eligibility, and state regulations. While commercial insurers may cover CAR-T therapy, they often require prior authorization, single case agreements and adherence to specific guidelines. Healthcare institutions must establish close collaboration with insurers to ensure proper reimbursement, especially considering that commercial insurance reimbursement rates can vary, necessitating negotiations on payment terms and prompt claims processing. It is important to prepare for navigating the prior authorization process and handling appeals, when necessary, with a focus on ensuring timely and accurate documentation of medical necessity to facilitate successful appeals. Additionally, the exploration of value-based agreements (VBAs) for CAR-T therapies, which align reimbursement with patient outcomes, could be considered. Value-based agreements encompass shared savings, risk-sharing, or pay-for-performance models, necessitating collaborative negotiations with payers.^36^ Some healthcare organizations have also examined bundled payment models, which encompass the entire episode of CAR-T therapy care, from infusion to post-treatment follow-up.^36^ Although these models can promote cost containment and care coordination, their successful implementation demands careful financial planning and effective collaboration with payers. Per the European Medicines Agency, CAR-T therapy falls under the designation of Advanced Therapy Medicinal Products (ATMP).^37^ In the European Union (EU), decision-making related to pricing and reimbursement is done at a national level and each country must make its own health technology assessment regarding effectiveness, safety, medical necessity, cost-effectiveness and budget impact.^37^ Outcome-based reimbursement and spread payment models are being increasingly used in the EU for CAR-T to help increase affordability.

To empower patients in making informed decisions, it is crucial to implement financial counseling services aimed at helping them understand their insurance coverage, copays, and available financial assistance options. Employing financial advocates can further aid patients in navigating the complex reimbursement landscape, identifying potential sources of financial support, and ensuring transparency regarding treatment costs, insurance coverage, and potential financial challenges. Moreover, many CAR-T manufacturers offer patient assistance programs designed to alleviate out-of-pocket costs for eligible patients, encompassing copays, deductibles, and various other expenses, such as local lodging and travel.

### Personnel Requirements

The implementation of outpatient CAR-T therapy programs requires the assembly of a highly skilled and specialized multidisciplinary team. This well-structured healthcare team is instrumental in ensuring the safe and effective delivery of patient care as CAR-T therapies transition to the outpatient setting. Key considerations regarding personnel requirements during this pivotal transition encompass:

1. Physicians: Physicians occupy a central role in patient selection, treatment planning, and ongoing care.
2. Advanced practice providers (APPs): Under the supervision of physicians, nurse practitioners and physician assistants are instrumental in conducting patient evaluations, contributing to treatment planning, and assisting with regular follow-up care.
3. Clinical pharmacists: Clinical pharmacists play a pivotal role in medication management, patient education, and adverse event management and are also routinely involved in CAR-T program policy and protocol development. At many institutions, clinical pharmacists also serve as the REMS program authorized representative.
4. Nurse navigators: Nurse navigators are responsible for orchestrating patient care coordination. They are vital in providing patient education, serving as an accessible point of contact for patients and caregivers throughout the entirety of the CAR-T therapy process.
5. Cell therapy specialists: These specialists often hold essential certifications and are trained in CAR-T therapy administration and management. Their responsibilities encompass the technical aspects of CAR-T product infusion. Coordinators also ensure the punctual scheduling of patient appointments, follow-up visits, and data collection.
6. Social workers: Providing indispensable psychosocial support, social workers assist patients and their families in navigating the emotional and practical challenges associated with CAR-T therapy. Their expertise extends to resource navigation, enabling them to connect patients with vital support services.
7. Financial counselors and patient advocates: Financial counselors are essential as they assist patients in comprehending their insurance coverage, costs, and potential avenues for financial assistance. Simultaneously, patient advocates navigate the healthcare system, addressing non-clinical barriers to care and ensuring patients receive the support they require.
8. Emergency response team: This specialized team is on standby for critical situations, including severe adverse events. Comprising critical care specialists, intensivists, and rapid response nurses, they are key to ensuring immediate intervention and facilitating patient transfer to a higher level of care, should the need arise.
9. Data managers and administrative staff: These professionals are entrusted with the collection, organization, and reporting of patient data, which serve critical functions in quality improvement initiatives and research endeavors. They also efficiently handle scheduling, patient communication, medical records, and various administrative tasks that underpin the seamless operation of outpatient CAR-T therapy programs. They may also serve as the REMS program authorized representative for the institution.
10. Cellular therapy technicians: These personnel are trained in the product chain of custody (collection, storage, handling, preparation, and transport) of CAR-T products and are key to maintaining product quality.

It is paramount to ensure that all personnel receive appropriate training and possess the necessary certifications pertaining to CAR-T therapy, thereby safeguarding patient safety. Effective collaboration among these diverse team members is indispensable for providing comprehensive patient care, vigilant monitoring of adverse events, and the attainment of successful outcomes in the outpatient CAR-T therapy setting. Furthermore, the pursuit of ongoing education and training is imperative to remain current with the latest advancements and best practices within the dynamic field of CAR-T therapy.

### Infrastructure Needs

The transition of CAR-T therapies to the outpatient setting necessitates the establishment of specialized infrastructure to facilitate the safe and efficient administration of these cutting-edge treatments.

1. Designated physical outpatient space: Key considerations include a dedicated treatment area tailored to accommodate CAR-T cell product infusions. This area must strictly adhere to infection control standards while providing comfortable seating arrangements for both patients and their caregivers. The treatment facility should meet all regulatory and accreditation requirements, particularly those concerning the handling of biological materials and patient privacy. Compliance with these standards ensures the utmost safety and quality of care.
2. In-house laboratory/transfusion services: The availability of an apheresis unit or collaboration with a nearby facility is pivotal for conducting leukapheresis procedures essential for collecting lymphocytes from patients. This unit should boast the requisite apheresis machines, a trained staff, and stringent quality control measures. Additionally, the facility must possess on-site laboratory capabilities to handle the processing, testing, and quality control of CAR-T products. Compliance with Good Manufacturing Practices (GMP) and other regulatory standards for cell processing is non-negotiable to ensure product integrity. Finally, the facility must be able to provide irradiated blood products for CAR-T patients who are pancytopenic.
3. Ancillary support systems and pathways: The facility should be well-equipped with emergency response tools, including crash carts, defibrillators, and advanced life support equipment. Clearly defined protocols for transferring patients to higher-level care units or facilities in the event of severe adverse events or complications that surpass the outpatient setting’s capacity are essential. Healthcare providers must receive thorough training in rapid response procedures and emergency protocols tailored to CAR-T therapy.
4. Electronic medical record support: Order sets, lymphodepleting treatment plan and supportive care plan builds, electronic flowsheets for documentation, and safety alerts all provide important patient care safeguards and facilitate standardization of care.

Given the growing availability and importance of remote patient monitoring and virtual services, the integration of telemedicine capabilities is a critical component. This infrastructure should align with regulatory and privacy requirements, facilitating virtual consultations with healthcare providers, particularly for patients residing at a distance from the treatment center.

The transition of CAR-T therapies to the outpatient setting necessitates a holistic approach to infrastructure development that prioritizes patient safety, regulatory adherence, and efficient care delivery. Collaboration with experts in facility planning, infection control, and regulatory affairs is imperative to ensure the establishment and maintenance of a suitable infrastructure tailored to the needs of outpatient CAR-T therapy programs.

### Standard Operating Procedures (SOPs)

The seamless transition of CAR-T therapies to an outpatient setting hinges on the development of comprehensive SOPs. These SOPs serve as the bedrock that guarantee safe, efficient, and uniform patient care. They encompass critical facets of CAR-T therapy administration and patient management. Some patients may not be eligible to receive their CAR-T therapy infusions in the outpatient setting. Institutions should establish precise eligibility criteria, including disease indications, performance status, organ function, prior treatments, and caregiver availability. The patient evaluation process should be clearly outlined and incorporate mandated laboratory tests, imaging, and specialist consultations to assess suitability for outpatient CAR-T therapy.

Patient and caregiver understanding and consent regarding treatment procedures, potential adverse events and significance of follow-up appointments are vital to the success of outpatient CAR-T therapy. Therefore, development of SOPs for acquiring informed consent, emphasizing a comprehensive understanding of CAR-T therapy-associated risks, benefits, and expectations is important. Protocols should be standardized but individualized to the patient’s treatment plan.

Stringent procedures governing CAR-T cell product receipt, storage, handling, and preparation, with meticulous adherence to manufacturer-specific requirements, should be established. The preferred regimens, dose modifications, administration guidelines, and supportive care should be clearly defined for the administration of lymphodepletion chemotherapy for each of the CAR-T products. The workflow for CAR-T cell infusion, with comprehensive patient monitoring during and post-administration, accompanied by explicit guidelines for addressing infusion-related adverse events should be described. The appropriate clinical/operational training and competencies for personnel, as well as the responsibilities of involved personnel, should also be clearly outlined in related policies.

Protocols for early recognition, assessment, and management of CAR-T therapy-related adverse events, including CRS and neurologic toxicities, should be defined. A structured schedule and frequency for patient monitoring, involving laboratory evaluations, imaging studies, and clinical assessments should also be established. This includes a comprehensive emergency response plan. Criteria for patient triage and the initiation of rapid response teams in cases of severe adverse events should also be standardized. Guidelines dictating patient discharge criteria, ensuring clinical stability and comprehensive patient/caregiver education regarding post-discharge care should be created to provide seamless communication and transitions of care.

To ensure quality assurance and regulatory compliance, institutions should employ regularly scheduled SOP reviews and updates, with competency assessments for healthcare providers and team members engaged in CAR-T therapy administration and patient care. Centers should confirm alignment of all SOPs with regulatory mandates and guidelines, including FDA-mandated REMS requirements.

SOPs represent indispensable tools for maintaining consistency, safety, and quality in transitioning CAR-T therapy to an outpatient milieu. Periodic SOP reviews and updates are imperative to adapt to evolving practices, ultimately enhancing patient outcomes while upholding the highest standards of patient safety.

### Metrics for Program Success

Establishing and monitoring key metrics is of paramount importance during the transition of CAR-T therapies to the outpatient setting. These metrics serve as critical evaluative tools, assessing the program’s effectiveness, patient outcomes, safety protocols, and resource utilization. Below are essential metrics deserving consideration:

1. Clinical outcomes including overall response rate (and complete response rates), progression-free survival, overall survival, and event-free survival
2. Adverse events including review of incidence and severity of CRS and neurotoxicity, as well as rates of infection and prolonged cytopenias
3. Patient experience with the completion of patient satisfaction surveys, patient-reported outcomes, and quality of life assessment
4. Resource utilization including assessment of infusion center/ambulatory clinic throughput, admission rates for adverse events, length of hospital stay (with associated costs), and frequency of patient ineligibility for CAR-T and/or need for inpatient cell infusion
5. Financial metrics including the cost per patient incorporating drug expenses, hospitalization and follow-up care, as well as reimbursement rates
6. Quality improvement metrics, including adherence to standardized protocols, time to CAR-T treatment, and protocol modifications
7. Patient access and equity metrics including geographic accessibility and monitoring for health disparities

Regularly collecting, dissecting, and acting upon these metrics constitute pivotal steps in optimizing the transition of CAR-T therapies to the outpatient setting. This approach assures patient safety, augments overall care quality, and necessitates the establishment of a data-driven culture. Leveraging electronic health records and data management systems facilitates metric tracking and reporting, contributing to the program’s continuous growth and improvement.

## Patient Selection Considerations

Thoughtful patient selection is crucial to maintaining a successful outpatient CAR-T program. Both disease and patient-related factors should be thoroughly evaluated to determine eligibility for outpatient treatment. Table 2 outlines key factors to consider when evaluating a patient for outpatient CAR-T therapy; however, additional criteria may be included based on the individual center’s resources and experience. Patients deemed ineligible for outpatient administration but remaining suitable candidates for CAR-T therapy should receive inpatient CAR-T infusion. These patients may also be considered for re-evaluation for outpatient therapy once the disqualifying issue has been resolved (e.g., resolution of infection or availability of appropriate caregiver) and bridging therapy can be used to control disease during this period. However, waiting to re-evaluate for outpatient administration should not be at the expense of delaying inpatient CAR-T infusion, if it is in the best interest of the patient, given their disease characteristics. Some centers also practice a pre-emptive admission approach for patients who are at higher risk for serious toxicity or adverse outcomes.^26,33^ This process involves administering lymphodepleting chemotherapy and CAR-T infusion in the outpatient setting and then admitting the patient either at first sign of grade 1 CRS or immune effector cell-associated neurotoxicity syndrome (ICANS), or at an otherwise pre-determined date, reflective of when toxicities most commonly initially occur.

**Table 2.** Outpatient CAR-T Patient Selection Considerations^31–33^

**Patient Considerations**	
Clinical eligibility	Disease burden Tumor volume/bulk Baseline inflammatory markers Platelet count Central nervous system involvement Performance status Cognitive ability Comorbidities Age Prior history of Cytokine Release Syndrome/Immune effector Cell Associated Neurotoxicity Syndrome with other immunoterapies
Social eligibility	Health literacy and compliance Availability of a reliable caregiver Health literacy and compliance Ability to provide transportation Ability to participate in/perform required monitoring Ability to recognize when to seek medical care

There are limited data available on outpatient CAR-T infusion in pediatric patients, and most centers still prefer to administer in the inpatient setting. Younger children require the use of alternative toxicity screening tools and increased reliance on their caregivers. However, older children and adolescents may reasonably be considered for outpatient therapy, and the same principles discussed herein would also apply in establishing such a program. Additional considerations may include additional protocols related to patient-specific monitoring and enhanced caregiver assessments and education, as well as the potential need for identification of multiple caregivers.

## Daily Outpatient Workflows

Prior to leukapheresis, the patient should receive thorough education of the CAR-T therapy process from the physician and nurse navigator, and be cleared by the various coordinating entities, such as finance and social work. By the start of lymphodepletion in the outpatient setting, a patient should receive counseling from the physician, APP, and/or clinical pharmacist on the chemotherapy, supportive medications, and adverse effects from the procedure. After laboratory and clinical evaluation of the patient, the provider may clear the patient to proceed with lymphodepletion. Tables 3 and 4 provide example daily outpatient workflows and admission pathways for patients undergoing outpatient administration of lymphodepletion and subsequent CAR-T therapy.

**Table 3.** Example Daily Outpatient CAR-T Workflow

Lymphodepletion (LD) stage (Day –6/-5 to Day –1)	Patient has completed provider assessment 1-2 days prior to LD and is cleared to proceed with treatment, and comprehensive patient/caregiver education and materials have been provided Team to ensure prescriptions have been sent to local pharmacy for supportive medications (e.g. infection and seizure prophylaxis) Patient presents to the outpatient day hospital/clinic daily for lab draw Provider or pharmacist provides patient and caregiver with wallet card and reinforces precautions, self-monitoring, and associated adverse effects RN patient assessment, lab review and clinical pharmacist review of LD chemotherapy regimen/dosing and supportive care, followed by pharmacist verification and preparation of LD chemotherapy Supportive therapies (hydration, tumor lysis syndrome prophylaxis, antiemesis medications) and administration of LD chemotherapy as per protocol RN reviews daily discharge criteria, and when appropriate, patient and caregiver are discharged to nearby housing
CAR-T administration (Day 0)	Patient presents to outpatient day hospital/clinic for lab draw Provider assesses patient and approves to proceed with CAR-T infusion RN coordinates with cellular therapy lab to determine time of product availability, and appropriate administration of pre-medications CAR-T product is administered per SOP following appropriate independent double-checks of CAR-T product RN performs and documents vital signs and pulse oximetry prior to pre-medications, at the start of infusion, every 15 minutes during, and end of infusion Patient is observed for a minimum of two hours post-infusion, and the RN/APP documents the daily CRS and ICANS evaluation Patient is set-up and educated on remote monitoring device, if applicable RN and designated physician/APP should be available to provide adverse event (CRS/ICANS or other AE) management and supportive care RN reviews daily discharge criteria, and hands off to patient and caregiver, reinforcing self-monitoring procedures and next steps upon experiencing symptoms
Post-infusion monitoring	Patient presents for labs and clinical assessment Daily clinic visits from day +1 to at least day +7 Visit frequency from day +7 to day +28 is determined by CAR-T product and provider/institution preference Some institutions may favor adding an evening telehealth follow-up visit in addition to the in-person visit Patient/caregiver hands off overnight updates and events to RN and/or APP RN reviews vital signs, performs neuro assessment and wellbeing assessment Provider assesses patient on outpatient CAR-T rounds RN provides supportive care measures per protocols as necessary (hydration, antimicrobials, electrolyte replacements, anti-emetics, etc.) RN reviews daily discharge criteria If not meeting discharge criteria, see Admission Pathways below

Abbreviations. LD, Lymphodepletion; CAR-T, Chimeric Antigen Receptor T Cell; SOP, Standard Operating Procedure; RN, Registered Nurse; APP, Advanced Practice Provider; CRS, Cytokine Release Syndrome; ICANS, Immune effector Cell Associated Neurotoxicity Syndrome; AE, Adverse Event;

**Table 4.** Example CAR-T Admission Pathways

**Admission Pathway Considerations**	
Triage Line: A designated 24-hour triage CAR-T line must be available for patients to report after-hours medical issues. The responding provider or RN would advise the patient in consultation with the designated on-call physician. 24-hour evaluation/triage center: The availability of a 24-hour triage center is recommended for the clinical evaluation of outpatient adverse events Inpatient bed availability: An ICU bed and a non-ICU bed should be available for the admission of a CAR-T patient experiencing adverse events requiring escalated care As per REMS, the appropriate staff should ensure the availability of tocilizumab (and other agents for the treatment of CRS) for either inpatient or outpatient administration	
**Example Admission Pathways**	
Outpatient day hospital/clinic → inpatient	If the patient is not suitable for discharge from the outpatient visit based on the institutional criteria, the designated provider will directly admit the patient (preferred) or have the patient evaluated for potential admission through the evaluation/emergency center as described below
Outpatient evaluation → inpatient	Patients are educated to report any infection-like or neurologic symptoms (including fever, neurological changes, respiratory distress) directly to the triage line Based on symptom severity and/or bed availability, the patient will be either proceed to the inpatient unit for direct admission or to the 24-hour emergency triage center/emergency room (ER) for further medical evaluation The designated rounding or on-call CAR-T physician will be contacted to discuss suspected or confirmed CAR-T related side effects The patient will be evaluated by a provider to determine the initial diagnostic and therapeutic interventions required, in a room appropriate for reducing infectious disease transmission If meeting an institution’s CAR-T admission criteria, the evaluating RN will accompany the patient on their transfer and provide verbal hand-off to the receiving inpatient RN
Life-threatening emergency	If the patient or his/her caregiver reports a life-threatening emergency, they will be instructed to activate the emergency medical system for transportation to the nearest emergency room They will also be instructed to request that the emergency room physician contact’s the institutions triage line, where the responding RN would facilitate communication between the ER and the designated on-call physician

Abbreviations. CAR-T, Chimeric Antigen Receptor T Cell; RN, Registered Nurse; ICU, Intensive Care Unit; REMS Risk Evaluation and Mitigation Strategy; CRS, Cytokine Release Syndrome; ER, Emergency Room;

## Conclusion and Future Directions

The future of CAR-T therapy is outpatient administration; however, the ability to do so requires careful planning to maintain patient safety. It also demands strict program oversight, adaptability, and quality metrics to optimize patient satisfaction and ensure cost-effectiveness. As we gain more real-world experience with outpatient CAR-T administration, it is expected that more community hospitals and hospital-affiliated offsite clinics will also begin providing these therapies. As a consequence, the distance to access a treating facility will be reduced, allowing more patients the opportunity to receive this crucial treatment without the need for temporary relocation. Furthermore, as cell therapy products expand to NK and other cell types, and as manufacturing processes expand to include in house or de-centralized manufacturing, the topic of outpatient delivery will remain pertinent. It is especially imperative that more outpatient programs are established as these products continue to move-up into earlier lines of therapy, as this will only increase the number of patients who need to access them.^3,4,9,12^ Additionally, the same infrastructure created for outpatient CAR-T administration has the potential for other applications, such as provision of the newly approved bispecific antibodies, which have a similar toxicity profile to CAR-T therapies. We find ourselves in a new era in the treatment of hematologic malignancies that necessitates adaptability in the ways we provide care to patients. Transitioning CAR-T to the outpatient setting is a critical step in that process.
